# Prolonging the time of progesterone supplementation to improve the pregnancy outcomes of single day 6 blastocyst transfer in frozen-thawed cycles: study protocol for a randomized controlled trial

**DOI:** 10.1186/s13063-022-07013-1

**Published:** 2022-12-19

**Authors:** Manlin Xu, Yuan Yan, Xiaoyue Shen, Haixiang Sun, Guijun Yan, Na Kong, Yue Jiang

**Affiliations:** 1 Center for Reproductive Medicine and Obstetrics and Gynecology, Nanjing Drum Tower Hospital, Nanjing University Medical School, Nanjing, China; 2 Center for Molecular Reproductive Medicine, Nanjing University, Nanjing, 210093 China

**Keywords:** Blastocyst, Frozen-thawed embryo transfer, Endometrial preparation, Randomized controlled trial

## Abstract

**Background:**

Infertility is one of the most important and underappreciated reproductive health problems in developing countries. Currently, in vitro fertilization and embryo transfer is the most effective treatment strategy for infertility. In a frozen-thawed cycle, single-blastocyst transfer can not only ensure relatively higher pregnancy and live birth rates but also effectively reduce the risk of maternal and neonatal complications. In frozen-thawed cycles, progesterone is initiated to promote the final phase of endometrial preparation prior to embryo transfer. However, the optimal duration of exposure to progesterone has remained inconclusive. Therefore, we designed a randomized controlled trial (RCT) to compare the effects of different prolonged progesterone transformation times (P+6 and P+7) on the pregnancy outcomes of D6 single blastocyst transfer in a frozen-thawed cycle.

**Methods:**

This is a single-center, prospective, randomized controlled clinical trial involving 900 patients with single blastocyst transfer in the frozen-thawed cycle, aged from 20 to 38 years, with less than three transfers, and with HRT-cycle single D6 blastocyst transfer in the current cycle. Participants will be randomly assigned (1:1) into two parallel groups: the transfer of day 6 blastocysts on the 7th day of progesterone supplementation and the transfer of day 6 blastocysts on the 6th day of progesterone supplementation. The primary outcome measure is the clinical pregnancy rate. Secondary outcome measures include the miscarriage rate and live birth rate.

**Discussion:**

This is the first randomized controlled trial to compare the transfer of day 6 blastocysts on the 6th and 7th day of progesterone supplementation. The results of this study will provide evidence for whether to prolong the duration of exposure to progesterone prior to embryo transfer.

**Trial registration:**

ClinicalTrials.gov, ID: NCT04938011. Registered on 19 June 2021.

**Supplementary Information:**

The online version contains supplementary material available at10.1186/s13063-022-07013-1.

## Administrative information

Note: the numbers in curly brackets in this protocol refer to SPIRIT checklist item numbers. The order of the items has been modified to group similar items (see http://www.equator-network.org/reporting-guidelines/spirit-2013-statement-defining-standard-protocol-items-for-clinical-trials/).

**Table:** 

Title {1}	Prolonging the time of progesterone supplementation to improve the pregnancy outcomes of single Day 6 blastocyst transfer in frozen-thawed cycles: study protocol for a randomized controlled trial
Trial registration {2a and 2b}.	Clinical Trials. gov, ID: NCT04938011.
Protocol version {3}	Registered on 19 June 2021.
Funding {4}	No funding was received.
Author details {5a}	^1^Center for Reproductive Medicine and Obstetrics and Gynecology, Nanjing Drum Tower Hospital, Nanjing University Medical School, Nanjing, China. ^2^Center for Molecular Reproductive Medicine, Nanjing University, Nanjing 210093, China.
Name and contact information for the trial sponsor {5b}	Nanjing University, Hankou Road 22, Gulou District, Nanjing, Jiangsu Province, China, Postcode: 210093
Role of sponsor {5c}	The sponsor played no part in study design; collection, management, analysis, and interpretation of data; writing of the report; and the decision to submit the report for publication.

## Introduction

### Background and rationale {6a}

Assisted human reproductive technology (ART) has undergone more than 40 years of development and has been increasingly used worldwide. The number of embryos transferred is often increased to improve clinical pregnancy rates, which also increases the risk of multiple pregnancies [1]. Therefore, single blastocyst transfer has become an important method to achieve safer ART. Compared with cleavage-stage embryos, blastocysts undergo a reselection process that spans the 8-cell-stage developmental block and eliminates embryos with poor developmental potential. Moreover, blastocyst transfer is more synchronized with endometrial development, and consequently, the pregnancy outcomes of fresh-cycle blastocyst transfer are superior to those of cleavage-stage embryos [2, 3]. Especially with the continuous improvement of vitrification technology, in a frozen-thawed cycle, single blastocyst transfer can not only ensure relatively higher pregnancy and live birth rate (LBR) but also effectively reduce the incidence of ovarian hyperstimulation syndrome, multiple birth rates, and fetal and maternal risk [4, 5].

The development rate of embryos from the cleavage stage to the blastocyst stage is not uniform, and blastocysts may form on days 5, 6, and 7 after fertilization. The implantation potential of blastocysts formed at different timing and its impact on pregnancy outcomes are still controversial [6]. In studies on fresh cycles, the pregnancy rate of D5 blastocysts was superior to that of D6 blastocysts, suggesting that the rate of blastocyst development predicts the implantation potential of the embryo [7, 8]. However, there is controversy regarding the clinical outcomes of D5 or D6 blastocyst transfer in frozen-thawed blastocyst transfer cycles. Frozen-thawed cycles ensure consistency of endometrial development and allow a better analysis of the impact of the blastocyst development rate on pregnancy outcomes. Recently, retrospective cohort studies concluded that the clinical pregnancy, implantation, and LBR after frozen-thawed blastocyst transfer are significantly higher in D5 embryos than in D6 embryos [9, 10]. However, some studies found no significant difference between day 5 and day 6 frozen-thawed blastocysts, with similar clinical pregnancy, implantation, and birth rates [11, 12].

Although the clinical outcomes of day 5 and day 6 frozen-thawed blastocyst transfers are still under discussion, this does not mean that the freezing of D6 blastocysts will be abandoned. For a proportion of patients with low ovarian function, poor embryo quality, the inability to tolerate another in vitro fertilization cycle, or other reproductive issues, the transfer of D6 blastocysts can still achieve a certain clinical pregnancy rate and thus a satisfactory pregnancy outcome, solving the patients’ dilemmas. The key to achieving successful implantation of frozen embryo transfer (FET) is the synchronous development of the embryo with the endometrium that shows embryo receptivity [13]. The human endometrium is receptive for only a relatively short period of time with strict temporal and spatial limitations, typically on days 20–24 of the menstrual cycle, which also corresponds to days 7–11 after the LH surge [14]. Compared with the D5 endometrium, the D6 endometrium is less receptive to embryos [15]. Thus, a proper assessment of the optimal endometrial preparation is the primary way to maximize the outcomes of FET [16].

Hormone replacement therapy (HRT) achieves artificial adjustment of the opening of the implantation window by mimicking the estrogen and progesterone changes in normal human menstrual cycles with exogenous hormones. Prolonging the time of endometrial transformation by progesterone in the FET cycle can reduce uterine contractions, enhance endometrial receptivity, and shorten the time interval between development and implantation after embryo implantation. Recent data from transcriptomic microarray and simple histologic endometrial dating have shown that delayed endometrial development in the luteal phase occurs in up to approximately 25% of the general population and, therefore, postponing the day of embryo transfer [17, 18]. However, existing studies of frozen-thawed blastocyst transfer cycles have focused more on the impact of different blastocyst qualities on pregnancy outcomes, and there is little research on the timing of D6 single blastocyst transfer. Currently, most blastocysts are usually transferred on the 6th day of progesterone supplementation, but the optimal duration of exposure to progesterone remains inconclusive. A recent RCT compared the outcomes of blastocyst transfer on the 5th or 7th day of progesterone administration, and the LBR tended to favor the 5th day of progesterone administration, although statistical significance was not shown [19]. However, a recent retrospective cohort study reported that day 6 blastocysts had nonsignificantly higher LBR when transferred on the 7th day of progesterone administration [20]. This randomized controlled study was conducted to compare the effects of different prolonged progesterone transformation times (P+6 and P+7) on the pregnancy outcomes of D6 single blastocyst transfer in frozen-thawed cycles.

### Objectives {7}

The objective is as follows: to compare the pregnancy outcomes by randomizing the transfer of a single day 6 blastocyst to the 6th day or 7th day of progesterone supplementation.

### Trial design {8}

This is a single-center, prospective, randomized controlled study. The type of this trial design is parallel group, it is also superiority trial, and the allocation ratio between the control group and the experimental group is 1:1.

## Methods: participants, interventions, and outcomes

### Study setting {9}

Patients are being recruited from the Affiliated Drum Tower Hospital of Nanjing University Medical School, China. This study has been approved by the ethics committees (listed in Additional file1).

### Eligibility criteria {10}

#### Inclusion criteria

1. Women aged 20 to 38 years
2. Those with less than three transfer cycles
3. Those undergoing an HRT frozen-thawed cycle with a single day 6 blastocyst transfer in the current cycle
4. Those who consent to participate in this study and sign the informed consent form

#### Exclusion criteria

1. A chromosomal abnormality in either the patient or their partner
2. Contraindications to HRT
3. Patients with intramural myoma affecting the morphology of the uterine cavity, severe adenomyosis, endometriosis, congenital uterine anomalies, endometrial tuberculosis, intrauterine adhesion, and other diseases significantly affecting embryonic implantation
4. Contraindications to HRT
5. Patients currently participating in other clinical studies

### Who will take informed consent? {26a}

Prior to study enrollment, study staff will provide participants with verbal and written information about the study in plain language. Participants will be given ample time to ask questions, and the couples will be asked to provide written consent if they agree to be part of the study.

### Additional consent provisions for collection and use of participant data and biological specimens {26b}

No additional consent will be collected.

## Interventions

### Explanation for the choice of comparators {6b}

The extended day is theoretically also covered by the implantation window. However, the results of the current D6 day embryo are not satisfactory, possibly because of the problem of synchronization and communication between the embryo and the endometrium. Additionally, the current tendency in assisted reproduction is to prolong the time of in vitro embryo culture to obtain more developed embryos, identify an implantation window for these mature embryos, and further increase the success rate of assisted reproduction.

### Intervention description {11a}

A total of 900 participants will be enrolled and equally randomized into two parallel treatment arms

A) Progesterone + 7 (P+7): the transfer of day 6 blastocysts on the 7th day of progesterone supplementation
B) Progesterone + 6 (P+6): the transfer of day 6 blastocysts on the 6^th^ day of progesterone supplementation

#### Endometrial preparation

All the included patients will undergo an HRT cycle for endometrial preparation. Treatment will start on day 2 or day 3 of menstruation with oral Femoston (estradiol 2 mg po tid). After approximately 10–12 days of estrogen treatment, a transvaginal ultrasound will be performed to measure the endometrial thickness. Two procedures for further treatment depending upon the endometrial thickness are listed as follows:

1. If the endometrium thickness is not less than 8 mm, patients will continue to take Femoston (estradiol tablet). On days 16–20 of the HRT cycle, patients will be changed over to receive oral Femoston (2 mg estradiol and 10 mg dydrogesterone tablet, po tid) and intramuscular injections of progesterone (60 mg qd) to transfer the endometrium into the secretory phase.
2. If the endometrium thickness is less than 8 mm, the dose of oral Femoston (estradiol tablet) will increase to 8 mg until the endometrium thickness reaches 8 mm. On days 18–20 of the HRT cycle, Femoston (2 mg estradiol and 10 mg dydrogesterone tablet, po tid) and progesterone (60 mg qd) will be administered to transfer the endometrium into the secretory phase. If the endometrium thickness is still less than 8 mm, it should be discussed with patients whether to proceed with FET or abandon the protocol.

#### Frozen-thawed embryo transfer

- *Group A*: the transfer of day 6 blastocysts on the 7th day of progesterone supplementation
- *Group B*: the transfer of day 6 blastocysts on the 6th day of progesterone supplementation

#### Corpus luteum support

Estradiol valerate will be stopped gradually after 8 weeks of FET; vaginal progesterone gel or oral dydrogesterone will be continued for 8 weeks of FET.

#### Follow-up evaluation

**Fig. 1:**
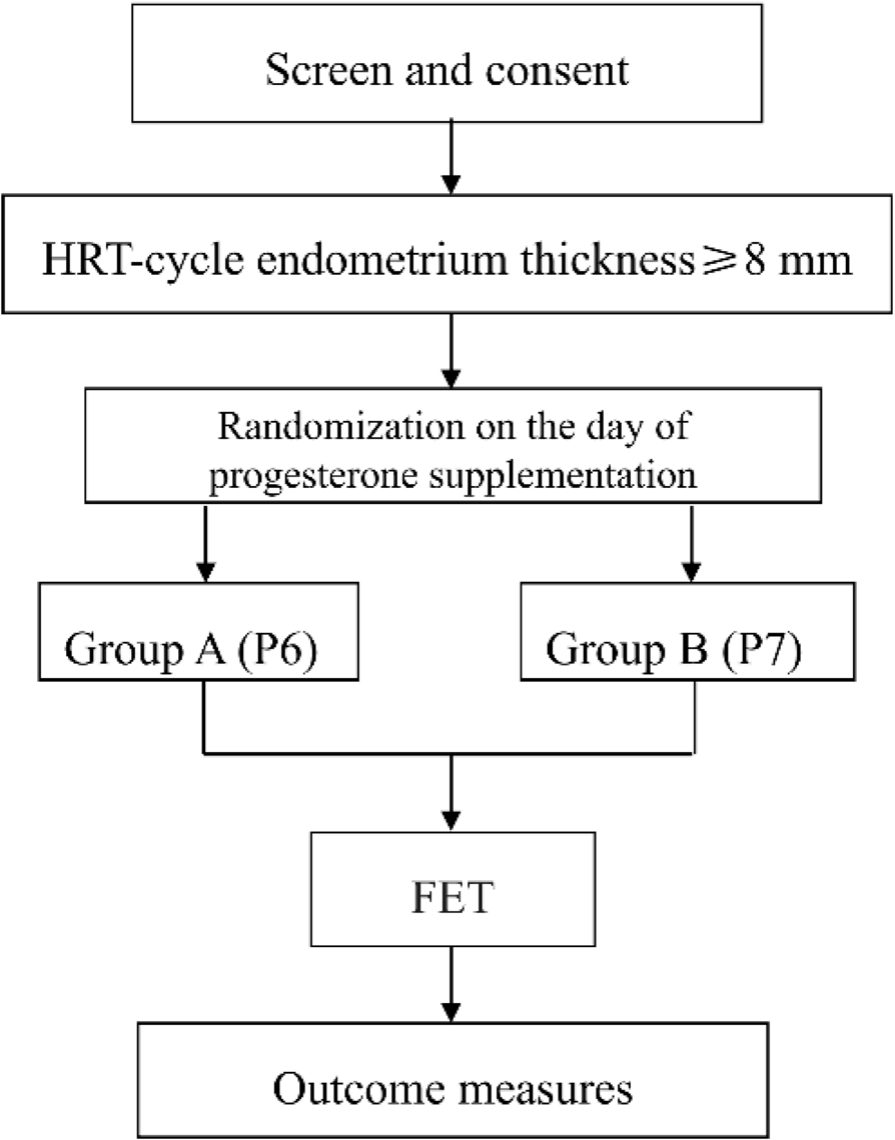
Study flowchart

1. Detection of serum β-hCG 12-14 days after embryo transfer
2. Ultrasound examination 30 days after embryo transfer to examine the intrauterine gestational sac with a fetal heartbeat. The flow chart of this study is shown in Fig.1.

### Criteria for discontinuing or modifying allocated interventions {11b}

#### Withdrawal criteria

Patients will not be suitable to continue the follow-up study if they have any of the following conditions:

1. Failure to comply with the program requirements for treatment
2. Unexpected withdraw from transplantation
3. The use of other drugs that may affect embryo implantation in the study

#### Suspension criteria

1. Patient demanding suspension
2. Safety reasons, such as treatment-related adverse events
3. Loss to follow-up or a lack of complete data

### Strategies to improve adherence to interventions {11c}

During the RCT, participants will receive an incentive of reduced costs for blood tests and ultrasound.

### Relevant concomitant care permitted or prohibited during the trial {11d}

Any treatment or change outside of the study intervention will be monitored throughout the trial.

### Provisions for post-trial care {30}

No special provisions are offered.

### Outcomes {12}

#### Primary outcome measure

The primary outcome measure is the clinical pregnancy rate: the ratio of the clinical pregnancy (intrauterine gestation sac with a fetal heart beat) number to the number of patients who received embryo transfer.

#### Secondary outcome measures

Secondary outcome measures are the miscarriage rate and LBR.

Miscarriage rate: The ratio of the number of nonviable pregnancies before 12 weeks of gestation to the number of clinical pregnancies.

LBR: The ratio of the number of normal fetuses delivered after 28 weeks of gestation to the number of patients who received embryo transfer.

### Participant timeline {13}

The participant timeline is shown in Table1.

**Table 1:** SPIRIT figure: schedule of enrollment, interventions, and assessments

Timepoint	Study period					
	Enrollment			Post-allocation		
	T1	T2	T3	T4	T5	T6
	Day of progesterone supplementation	Period of HRT-cycle	Day of embryo transfer	14 days after transfer	30/42 days after transfer	After delivery
**Enrollment:**						
Eligibility screen	X					
Informed consent	X					
Randomization	X					
Basic information login	X					
Physical examination	X					
Medical/treatment history	X					
Pelvic ultrasound		X			X	
Serum E2, P level		X				
**Intervention:** Embryo quality			X			
**Data collection:**						
Serumβ-hCG level				X		
Primary outcome measure					X	
Secondary outcome measures					X	X
Compliance assessment		X	X			
Adverse events		X	X			
Medical record review					X	X

### Sample size {14}

The sample size calculation is based on the clinical pregnancy rate. Retrospective data analysis (from January 2019 to December 2020, a total of 490 cycles of D6 single blastocyst transfer) showed that the clinical pregnancy rate of D6 single blastocyst transfer was approximately 49%. In our present study, we hope to improve the clinical pregnancy rate to 60%, the two-sided significance level will be set at *α* = 0.05, and the statistical power will be calculated as 1 − *β* = 0.90. The ratio between groups will be 1:1. The number of samples required for each group is 430. According to the 5% loss rate, there should be no fewer than 450 participants in each group, for a total of 900 participants.

### Recruitment {15}

Potential participants will be recruited from Nanjing Drum Tower Hospital (Nanjing, China), which is qualified for FET. Our study will be propagated via the internet, bulletin boards, and posters in hospitals, with a hotline provided for potential volunteers to call. Eligible patients from the outpatient department will be asked to talk face to face with researchers and will be given detailed information about the study. If patients are eligible and interested in participating, they will be asked to sign an informed consent form. The recruitment period is from July 2021 to June 2024, and the estimated recruitment rate is more than 95%.

## Assignment of interventions: allocation

### Sequence generation {16a}

A 1:1 allocation to either group A or group B will be generated by Stata 15.0 (SPSS Inc., Chicago, IL, USA) and will be used for randomization.

### Concealment mechanism {16b}

The use of Stata to randomize using a password restricted website will ensure concealment.

### Implementation {16c}

Randomization was implemented by only one research assistant who is not involved in the trial in any way. Participants will be informed about their allocation via mail.

## Assignment of interventions: blinding

### Who will be blinded {17a}

This study is an open trial. Both patients and researchers know the group assignments in the trial and only the statisticians are blinded to the grouping.

### Procedure for unblinding if needed {17b}

No procedures are needed for unblinding until the finalization of the main data analysis.

## Data collection and management

### Plans for assessment and collection of outcomes {18a}

The time points of patient recruitment, intervention, data collection, processing, and follow-up operations are described in Table1. The collected data will be entered into the electronic data system of our reproductive medicine center.

### Plans to promote participant retention and complete follow-up {18b}

There is no additional incentive for participants to complete the trial. Based on our experience, participants are usually grateful for any research conducted due to the strong reproductive requirements.

### Data management {19}

All study staff, including outcome assessors and statisticians, will be well trained about data management. The collected data will be entered into the electronic case report form of our reproductive medicine center and stored on a secure server. The private information of patients (name, telephone number, and ID number) will be kept anonymous to ensure participant confidentiality. The clinical research associates are responsible for verifying the accuracy of the data and ensuring that the information will not be disclosed.

### Confidentiality {27}

Paper files will be kept in a locked filing cabinet in the treating hospital. Electronic documents will be stored in a password-protected computer, with access restricted to the principal investigator. All research documents will be preserved for at least 5 years after publication.

### Plans for collection, laboratory evaluation, and storage of biological specimens for genetic or molecular analysis in this trial/future use {33}

Not applicable—no identifying images or other personal or clinical details of participants are presented here or will be presented in reports of the trial results. Informed consent materials are available from the corresponding author on request.

## Statistical methods

### Statistical methods for primary and secondary outcomes {20a}

SPSS 13.0 software was used for analysis. The *χ*^2^ test will be applied to detect the differences in counting data (such as the clinical pregnancy rate, implantation rate, and abortion rate). Comparison of the measurement data (such as age, the basic FSH level, the AFC, Gn dosage and days, the number of eggs taken, and the fertilization rate) between groups will be performed using an independent sample *t* test. A *P* value < 0.05 will be considered statistically significant.

### Interim analyses {21b}

No interim analyses are planned.

### Methods for additional analyses (e.g. subgroup analyses) {20b}

No additional analyses are planned.

### Methods in analysis to handle protocol non-adherence and any statistical methods to handle missing data {20c}

Data will be analyzed following the ITT principle. Missing data will be analyzed. The imputation of missing values is not intended, although multiple imputations might be used within sensitivity analyses assuming data are missing at random.

### Plans to give access to the full protocol, participant level-data and statistical code {31c}

There is no additional protocol to this version. Any data, statistical code, or documentation may be provided by the corresponding author upon request.

## Oversight and monitoring

### Composition of the coordinating center and trial steering committee {5d}

Since this is a single-center trial, a formal coordinating center is not deemed necessary. The study team meets weekly. Principal investigators will join the meeting monthly.

### Composition of the data monitoring committee, its role and reporting structure {21a}

Given the small size of our monocentric trial with few researchers involved and without funding, there is no need for an additional data monitoring committee.

### Adverse event reporting and harms {22}

Adverse events (AEs) are defined as any untoward or unfavorable medical occurrences associated with the subject’s participation during the research. AEs in this study will be divided into study-related AEs and non-study-related AEs, which will be judged by clinicians at the time of recording. AEs related to the study will include hepatic injury and thrombosis. AEs related to Femoston and progesterone include nausea, dizziness and headache, fatigue, measles, breast swelling, menorrhagia or amenorrhea, hepatic dysfunction, edema, and weight gain.

All AEs will be recorded in detail. Once any adverse reaction occurs during medication use, the drug will be stopped immediately. Serious AEs will be reported to the principal investigator immediately, and appropriate measures will be initiated instantly. The ethics committee will determine whether the AE is likely to have been associated with the experimental drug and whether it is necessary to break the blinding codes.

### Frequency and plans for auditing trial conduct {23}

The whole trial progress will be monitored by the data management staff. Any data inconsistencies, missing data, and time window violations will be reported. The process will be independent from investigators and the sponsor.

### Plans for communicating important protocol amendments to relevant parties (e.g. trial participants, ethical committees) {25}

Any changes or amendments in protocol will be reviewed by the all investigators and be approved by local ethic committee. Any modifications will be eventually reported to the trial registration center, and the trial protocol will be updated.

### Dissemination plans {31a}

The results of the study will be disseminated unreservedly. After this study is finished, we are expected to publish one or more scientific manuscripts in peer-reviewed journals. The study data will be present at national and international conferences for further dissemination.

## Discussion

This is a trial comparing the effects of different progesterone administration times (P+6 and P+7) on the pregnancy outcomes of D6 single blastocyst transfer in frozen-thawed cycles. We plan to enroll 900 subjects from the Affiliated Drum Tower Hospital of Nanjing University Medical School. Enrollment began in July 2021. At the time of manuscript preparation, more than 110 subjects were enrolled. The results of this large prospective, RCT will clarify the best timing for the use of progesterone in D6 blastocyst transfer and improve the pregnancy rate of D6 blastocysts.

In HRT cycles, progesterone is initiated to promote the final phase of endometrial preparation prior to embryo transfer. In a previous study at our center, 151 patients with hormone replacement frozen-thawed cycle blastocyst transfer were randomly divided into two groups. The results suggested that blastocyst transfer on the 7th day of progesterone intimal transformation in the HRT frozen-thawed cycle had a higher clinical pregnancy rate than that on the 6th day. Especially for D6 single blastocysts, appropriately delaying the transfer time may be a way to improve the outcomes of frozen-thawed embryo transfer.

The literature mainly focuses on the ideal route of administration and dose of progesterone supplementation, and it is difficult to reach any consistent conclusion with the limitation of the retrospective study design. Therefore, a well-designed, prospective, randomized trial is urgently required to inform physicians as to whether to prolong the duration of exposure to progesterone prior to embryo transfer.

### Trial status

The study was conceived and designed in 2021. This protocol version was approved by the Affiliated Drum Tower Hospital of Nanjing University Medical School. It was registered on 19 June 2021, and the registry number is NCT04938011 (https://register.clinicaltrials.gov). The recruitment period is from July 2021 to June 2024.

## Supplementary Information

**Additional file 1.**

## Data Availability

All data generated or analyzed during this study are included in this article. Further enquiries can be directed to the corresponding author.

## Abbreviations

ART: Assisted reproductive technology
LBR: Live birth rate
FET: Frozen embryo transfer
HRT: Hormone replacement therapy
RCT: Randomized controlled trial
AE: Adverse event
hCG: Human chorionic gonadotrophin
LH: Luteinizing hormone
FSH: Follicle-stimulating hormone
