# Supplementary material for: Prolonging the time of progesterone supplementation to improve the pregnancy outcomes of single day 6 blastocyst transfer in frozen-thawed cycles: study protocol for a randomized controlled trial
Source: Trials. 2022 Dec 19;23:1024. doi: 10.1186/s13063-022-07013-1 (PMC9764536; doi:10.1186/s13063-022-07013-1)
Supplement: Supplementary file 1 — Additional file 1. [file 13063_2022_7013_MOESM1_ESM.docx]

NO.AF/SC-08/03.0

Nanjing Drum Tower Hospital

Ethics Committee Approval

IRB Review Approval Letter

NO.2021-177-01

| Title of Project | Prolonging the time of progesterone supplementation to improve the pregnancy outcomes of single Day 6 blastocyst transfer in frozen-thawed cycles: study protocol for a randomized controlled trial. | | |
| --- | --- | --- | --- |
| Protocol ID | NA | Study Start and Completion | July 2021-June 2024 |
| Application/CRO Company | None | | |
| Responsible Party | Nanjing Drum Tower Hospital | Study acceptance No. | NA |
| Department | Obstetrics and Gynecology | Principal Investigator | Yue Jiang |
| Phases of Clinical Trials | □Phase I □Phase Ⅱ □Phase Ⅲ □Phase Ⅳ √Scientific research | | |
| Review Approach | √Meeting Review  □Expedited Review | Review Type | □First review  □Second review  □ Follow-up review |
| Review Date | 2021-05-13 | Review Place | Meeting room on the 9th floor of Building 2 |
| Chief Reviewer | See the meeting sign-in sheet for details | | |
| Documents for Ethical Review: | | | |
| 1. Initial review application 2. Clinical study protocol (Version No: SZ-2021-ETP7-1, Version Date: 2021-04-20) 3. Informed consent (Version No: SZ-2021-ETP7-1, Version Date: 2021-04-20) 4. Materials for recruiting subjects (Version No: SZ-2021-ETP7-1, Version Date: 2021-04-20) 5. Case Report Form (Version No: SZ-2021-ETP7-1, Version Date: 2021-04-20) 6. Investigator's brochure (Version No: SZ-2021-ETP7-1, Version Date: 2021-04-20) 7. CV of main researchers 8. GCP certificate | | | |
| Review comments of the Ethics Committee | | | |
| Statement:  1. This letter of consent is valid for three years following the date of its initial assessment and approval. Please keep requesting an extension of the letter of consent's validity period if it has passed its expiration date.  2. The projects must adhere to the guidelines established by this ethical commission. This ethical committee operates in accordance with the Helsinki Declaration, ICH-CCP, and the State Drug Administration's Quality Management Specifications for Drug Clinical Trials. the requirements of other pertinent national laws and regulations, the National Health Commission's Measures for Ethical Review of Biomedical Research Involving Human Beings, and the Quality Management Specifications for Clinical Trials of Medical Devices.  3. Any alteration to the predetermined clinical study plan, informed consent, or other documents, as well as the main investigator's replacement. Before making any changes, please kindly submit an application to the Ethics Committee in a timely manner and have their written approval.  4. Please inform the sponsor and the Ethics Committee in time of adverse events and unexpected events that affect the risk benefit ratio of the study.  5. If the applicant applies to terminate/suspend the agreed research, please submit the application to the Ethics Committee in advance.  6. Restarting Terminate or suspend the agreed research, and submit the restart application before starting. | | | |
